# An aorto–left atrial fistula as a fatal complication of radiofrequency ablation for atrial fibrillation: a case report

**DOI:** 10.1093/ehjcr/ytag389

**Published:** 2026-05-29

**Authors:** Krystian Krzyżanowski, Agata Krzesiak, Marek Kiliszek

**Affiliations:** Department of Cardiology and Internal Diseases, Military Institute of Medicine—National Research Institute, Szaserow 128, Warsaw 04-141, Poland; Cardiology Department, Cardinal Stefan Wyszyński Voivodeship Hospital in Łomża, Al. Piłsudskiego 11, Łomża 18-404, Poland; Department of Cardiology and Internal Diseases, Military Institute of Medicine—National Research Institute, Szaserow 128, Warsaw 04-141, Poland

**Keywords:** aorto-left atrial fistula, Atrial fibrillation, Ablation, Case report

## Abstract

**Background:**

Aorto–atrial fistulas are extremely rare and potentially fatal complications of cardiac procedures.

**Case report:**

An 81-year-old woman with persistent AF underwent radiofrequency ablation with additional anterior wall lesions for extensive low-voltage areas. Seven days later, she was readmitted with non-specific symptoms. Despite initial improvement with pharmacological therapy, she developed sudden haemodynamic collapse. Echocardiography revealed an aorto–left atrial fistula. Intensive treatment was unsuccessful, and the patient died.

**Discussion:**

This case illustrates a rare, fatal complication of anterior wall ablation for atrial fibrillation. Awareness of this risk is essential, especially in elderly or frail patients requiring extensive ablation.

Learning points

- An aorto-left atrial fistula is an exceedingly rare, life-threatening complication of atrial fibrillation ablation.
- The segment of the left atrial wall adjacent to the posterior aspect of the aorta is the thinnest portion of the atrial myocardium, and extensive ablation with a high Ablation Index in this area may pose a risk of aorto–atrial fistula.

## Background

Aorto-atrial fistulas are exceedingly rare conditions representing abnormal communication between the ascending aorta and either the right or left atrium. The aetiology of aorto-atrial fistula is diverse, with infective endocarditis being the most common cause.^1^ Other aetiologies include aortic aneurysm, vasculitis, trauma, and congenital defects. In rare cases, the condition is iatrogenic, occurring as a complication of cardiac surgery or transcatheter interventions. The clinical presentation of an aorto-atrial fistula varies widely. Some patients remain asymptomatic, with the condition discovered incidentally. Others may present with palpitations, dyspnoea, chest pain, fatigue, or cough. In advanced cases, the fistula can lead to overt heart failure or recurrent respiratory infections. We present a rare case of an aorto–left atrial fistula occurring as a fatal complication of radiofrequency ablation for atrial fibrillation.

## Summary figure

**Figure:**
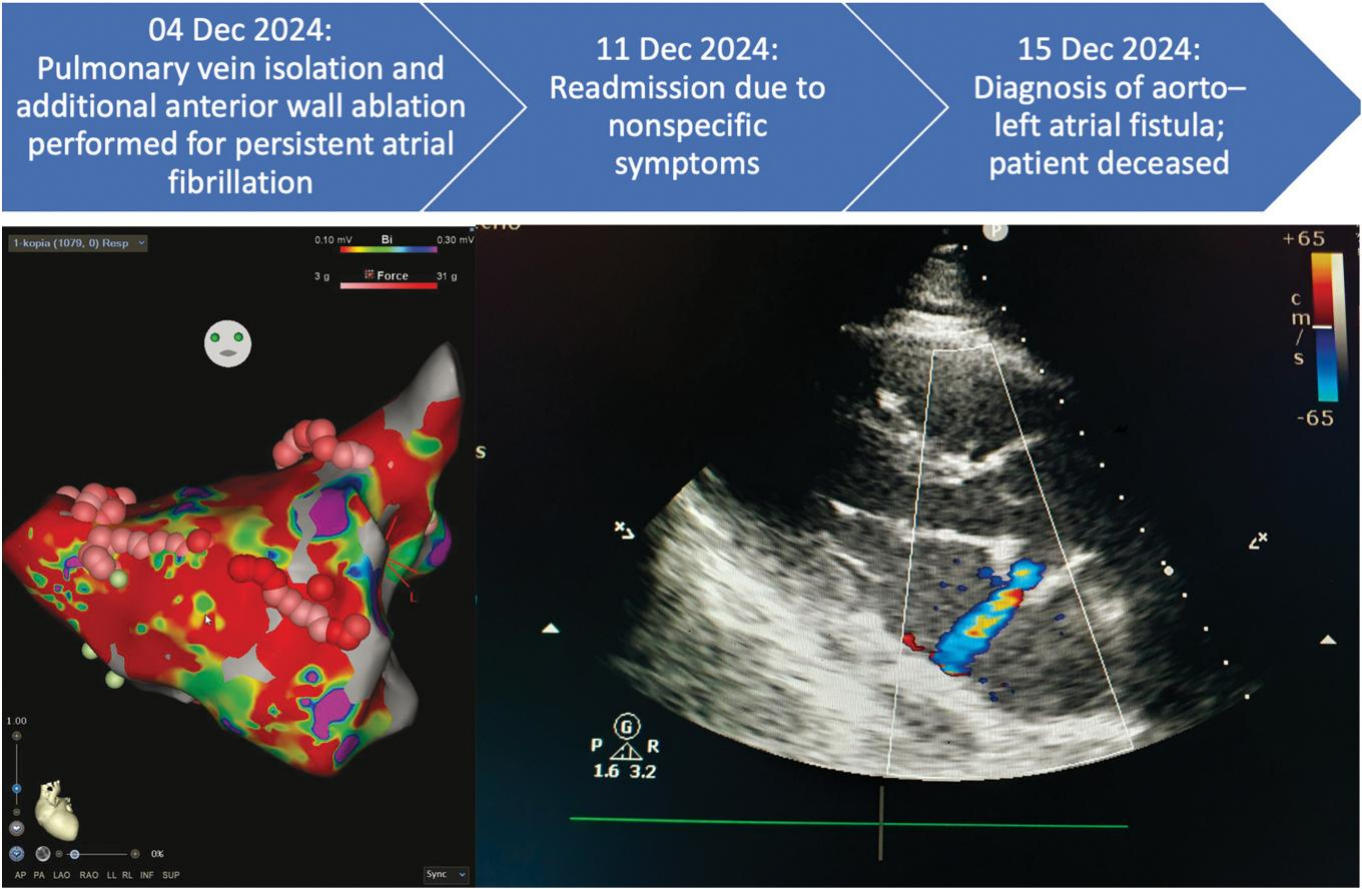


## Case presentation

An 81-year-old woman with persistent atrial fibrillation was admitted to our clinic for catheter ablation. She had a 1-year history of persistent atrial fibrillation, EHRA 2, and chronic heart failure with preserved left ventricular ejection fraction, NYHA II. Electrical cardioversion had previously been attempted, but arrhythmia recurred within several minutes. Transthoracic echocardiography showed a mildly enlarged left atrium, preserved left ventricular systolic function, and no significant valvular abnormalities. The patient’s other comorbidities included arterial hypertension, a remote ischaemic stroke, and chronic kidney disease.

**Figure 1:**
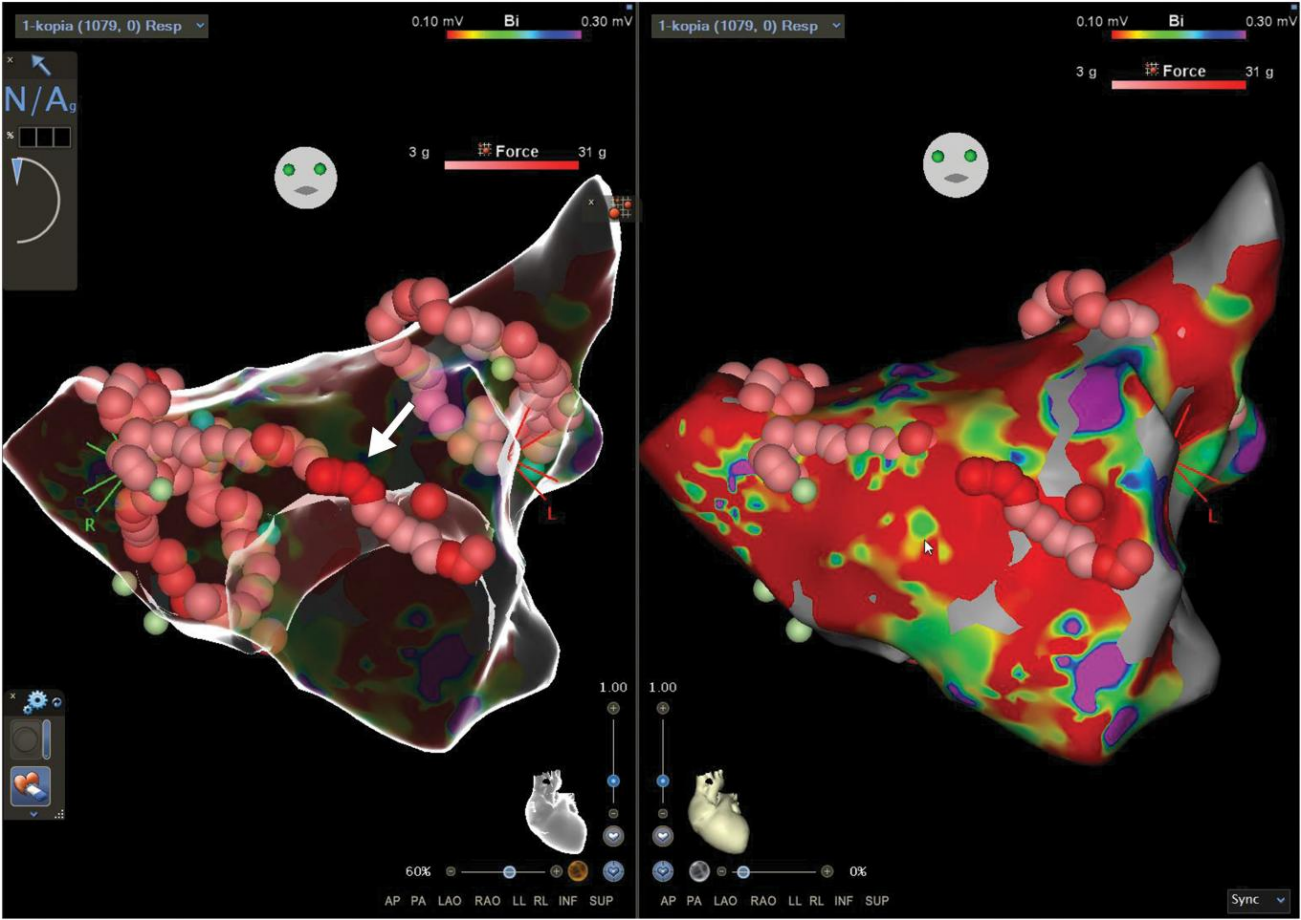
Endocardial 3D voltage map in anteroposterior view. (*A*) Anterior linear ablation line with point tags indicating maximal contact force; the possible site of perforation is marked by an arrow. (*B*) Extensive low-voltage areas visible along the anterior wall.

Radiofrequency catheter ablation was performed under conscious sedation. Left atrial access was obtained via a double transeptal puncture, guided by continuous pressure monitoring and fluoroscopy. A circumferential mapping catheter (Lasso 15 mm, spacing 4,5 mm, Biosense Webster, Irvine, CA, USA) was used for mapping, and an irrigated contact force catheter (Navistar ThermoCool SmartTouch, Biosense Webster, Irvine, CA, USA) was used for ablation. Catheter navigation was guided by fluoroscopy in combination with a three-dimensional electroanatomical mapping system (CARTO 3, Biosense Webster, Irwindale, CA, USA). Detailed left atrial mapping during atrial fibrillation demonstrated extensive low-voltage areas, predominantly on the anterior wall. Ipsilateral pulmonary veins were successfully isolated at first-pass. An anterior linear ablation was created between the mitral annulus and the right superior pulmonary vein. Radiofrequency power was limited to 40 W. The ablation index threshold was set at 500 for the anterior wall and 400 for both the roof and posterior wall. The maximal contact force was 31 g (*Figure 1*). Successful electrical cardioversion was performed, but atrial fibrillation recurred before the patient was discharged.

**Figure 2:**
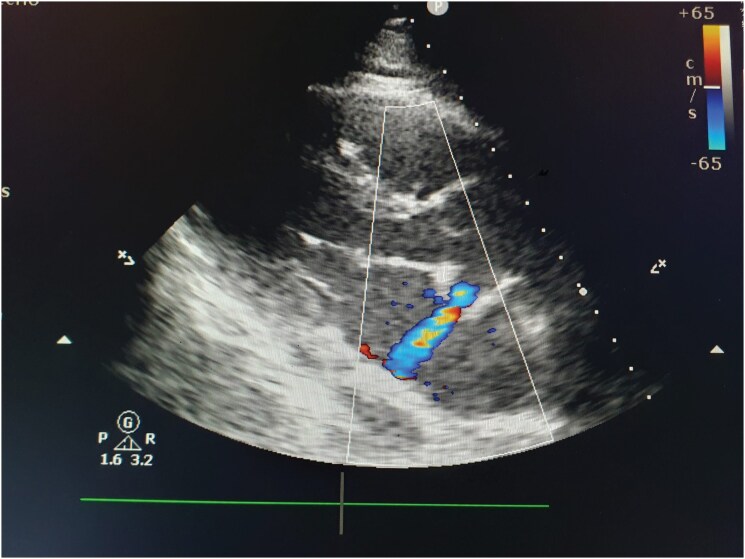
Colour-flow Doppler image demonstrating abnormal flow between the ascending aorta and the left atrium.

**Figure 3:**
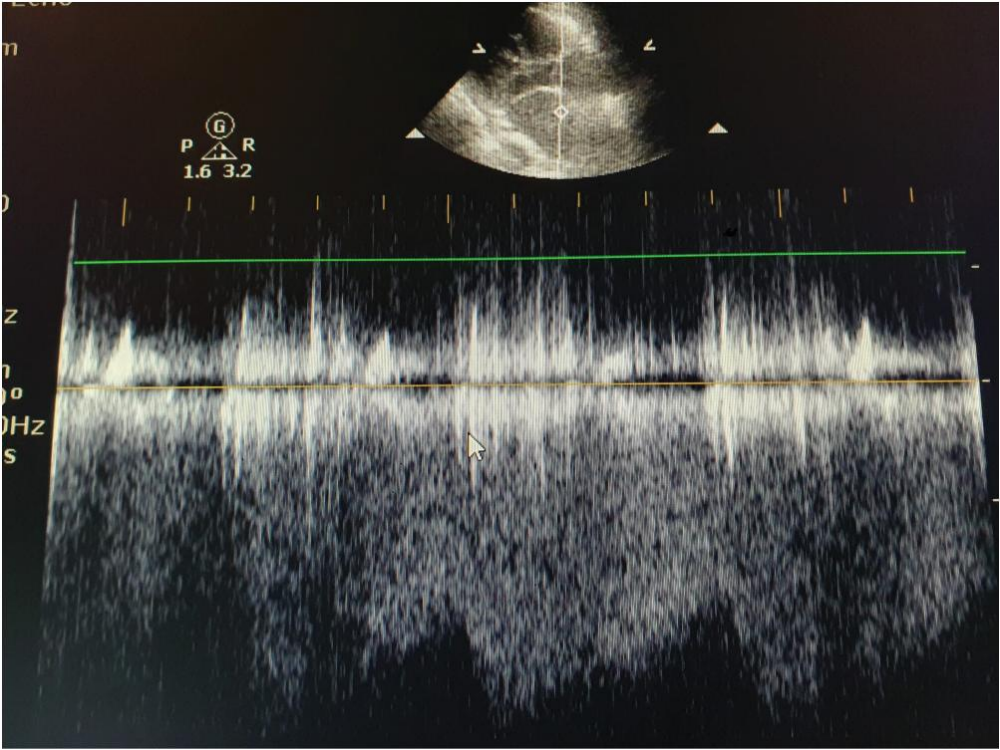
Continuous-wave Doppler recording confirming flow between a high-pressure and a low-pressure chamber.

Seven days after the ablation, the patient was admitted with fever, dyspnoea, and general malaise. She was in sinus rhythm, and her blood pressure was within normal limits. Auscultation revealed crackles at the bases of both lungs. Chest radiography demonstrated congestive-inflammatory consolidations in the lower and perihilar lung regions. Empirical antibiotic therapy was initiated, and initial clinical improvement was observed. On the fifth day of hospitalization, the patient developed generalized weakness, worsening dyspnoea, and non-specific discomfort in the chest and upper abdomen. Physical examination revealed epigastric tenderness without other significant findings. Electrocardiography showed atrial fibrillation with a ventricular rate of 90–100 beats per minute. During an abdominal computed tomography scan, the patient experienced sudden haemodynamic collapse and cardiac arrest, from which she was successfully resuscitated. Subsequent transthoracic echocardiography revealed an aorto-left atrial fistula (*Figures 2* and *3*). Despite mechanical ventilation and intensive pharmacological support, progressive haemodynamic deterioration ensued, and the patient died.

## Discussion

Pulmonary vein isolation remains the cornerstone of catheter ablation for both paroxysmal and persistent atrial fibrillation.^2^ However, the efficacy of pulmonary vein isolation alone in patients with persistent atrial fibrillation is limited. Various adjunctive ablation strategies have been proposed for this patient population, but none has been consistently proven to be superior.^3^ One such approach involves identification and ablation of low-voltage areas within the left atrium, which are considered surrogate markers of atrial fibrosis––a potential substrate for arrhythmia initiation and maintenance. The results of studies assessing the efficacy of low-voltage–guided ablation have been conflicting,^4–6^ although some have demonstrated improved outcomes compared with pulmonary vein isolation alone.^4^ In the present case, the patient had extensive areas of low-voltage areas, particularly along the anterior wall. Therefore, additional linear ablation along the anterior wall was performed in conjunction with pulmonary vein isolation. The ‘PVI plus’ strategy during the initial procedure in patients with persistent atrial fibrillation remains controversial, although it has been adopted by some centres.^3,4^ A limitation of the present case study is the use of a circumferential mapping catheter with an interelectrode spacing of 4.5 mm, rather than a high-resolution mapping catheter, and the fact that mapping was performed during atrial fibrillation. However, overestimation of the anterior scar is unlikely, as an extensive area with bipolar voltage below 0.1 mV was identified.

To date, only three cases of aorto-atrial fistula following atrial fibrillation ablation have been reported in the literature. In two of these, an aorto–right atrial fistula developed as a result of transseptal puncture during the ablation procedure.^7,8^ Both patients were asymptomatic with regard to the fistula, which was diagnosed using echocardiography and computed tomography. Surgical suture closure was successfully performed in each case. In the third case, radiofrequency ablation for atrial fibrillation (extent not specified) was complicated by a moderate pericardial effusion.^9^ Transthoracic and transoesophageal echocardiography revealed abnormal flow from the non-coronary cusp of the aortic valve into the left atrium. Percutaneous closure using an Amplatzer occluder was successfully performed.

In our case, the fistula developed as a result of ablation along the anterior wall, since the aortic shunt involved the left atrium and the transseptal puncture was uneventful. The clinical presentation was initially subtle, with non-specific symptoms that delayed diagnosis. Subsequently, the condition progressed rapidly, resulting in haemodynamic deterioration and death. A retrospective analysis of the ablation protocol did not reveal any deviations from the standard strategy routinely employed at our centre for patients with low-voltage areas. The use of 40 W power settings and an Ablation Index >500 on the anterior wall in the present case appears to be within acceptable limits.^3,10^ However, left atrial myocardial thickness is heterogeneous, averaging 4.5 mm in the superior wall, 3.9 mm in the lateral wall, and 3.3 mm in the anterior wall. The region of the anterior wall located posterior to the aorta may become markedly thin, measuring as little as 1.5 mm.^11^ It is noteworthy that the patient in the present case was of advanced age and mildly frail. The combination of these features, together with extensive ablation, warrants consideration when evaluating the risk of such rare complications.

Despite the best efforts of the authors, consent for publication was not obtained for this case. Every effort has been made to anonymise the case. The situation has been discussed with the editor.

## Supplementary Material

ytag389_Supplementary_Data

## Data Availability

The data underlying this article will be shared on reasonable request to the corresponding author.

## Lead author biography

Dr Krystian Krzyzanowski is a cardiac electrophysiologist at the Military Institute of Medicine in Warsaw, Poland. He graduated from the Medical University of Warsaw in 2006. His main clinical and research interest is catheter ablation of atrial fibrillation.
